# Exploration of the utility of different doses of Qingfei Dayuan granules: a multicenter, randomized, double-blind, placebo-controlled trial

**DOI:** 10.18632/aging.205601

**Published:** 2024-02-26

**Authors:** Xiaoyun Zhu, Weinan Li, Yi Yang, Hengfei Li, Min Yang, Jilong Zhang, Xucheng Li, Guangjun Yan, Xiongfei Wu, Weijun Zhao, Mengdi Cui, Xi Yang, Xinyu Hu, Juan Huang, Yuanming Ba

**Affiliations:** 1 Hubei University of Chinese Medicine, Wuhan, China; 2 Hubei Provincial Hospital of Traditional Chinese Medicine, Wuhan, China; 3 The Affiliated Hospital of Hubei University of Chinese Medicine, Wuhan, China; 4 Hubei Provincial Academy of Traditional Chinese Medicine, Wuhan, China; 5 Hubei University of Chinese Medicine, Department of Preventive Medicine, School of Basic Medicine, Wuhan, China; 6 Wuhan NO.1 Hospital, Fever Clinic, Wuhan, China; 7 Wuhan Hospital of Traditional Chinese Medicine, Emergency Department, Wuhan, China; 8 Jingzhou Hospital of Traditional Chinese Medicine, Jingzhou, China; 9 People’s Hospital of Hanchuan, Department of Infectious Diseases, Hanchuan, China; 10 Yichang Hospital of Traditional Chinese Medicine, Respiratory Department, Yichang, China

**Keywords:** Qingfei Dayuan granules, dose-effect relationship, different doses, Chinese medicine granules, trial

## Abstract

Background: Clinical studies have confirmed that Qingfei Dayuan (QFDY) granules are effective in the treatment of influenza and upper respiratory tract infections (URTIs) caused by pulmonary heat-toxin syndrome (PHTS). Granules of Chinese medicine formulations have become a widely used dosage form in clinical practice. With the continuous optimization of extraction technology, the advantages of Chinese medicine granules have been gradually demonstrated, but the price of Chinese medicine granules is generally higher than that of traditional dosage forms of Chinese medicine, and we support the rational use of the appropriate dosage of QFDY for patients with these conditions. Therefore, we set up half of the conventional dose as the low dose group, and designed the three-arm study to rigorously compare the efficacy difference of low-dose QFDY, QFDY and the placebo group, with the expectation of providing scientific support for the rational selection of the dose and the safe and effective use of the medicine in clinical practice.

Methods: We recruited 108 patients with clinical diagnoses of influenza and URTIs caused by PHTS to receive treatment at six hospitals in Hubei, China. Using a centralized randomization system, patients were randomly assigned at a 1:1:1 ratio to the QFDY, low-dose QFDY, or placebo control groups to receive the corresponding drug, and the study physicians, subjects, outcome assessors, and statisticians were unaware of group assignments. The primary outcome was the time to complete fever relief. Secondary outcomes included the efficacy of Chinese medicine in alleviating signs and symptoms and the disappearance rate of individual symptoms. Adverse events were monitored throughout the trial.

Results: A total of 108 patients were recruited. A total of 106 patients were included in the full analysis set (FAS). In the FAS analysis, there was no statistically significant difference in baseline of the three groups before treatment (P > 0.05).

1. Regarding the median time to complete fever relief, the QFDY, low-dose QFDY and placebo groups had median times of 26 h, 40 h and 48 h, respectively. The QFDY group had a shorter time to complete fever relief than the placebo group, and the difference was statistically significant (P < 0.05), while the low-dose QFDY group had a shorter time than the placebo group, but the difference was not statistically significant (P > 0.05).

2. In terms of the total efficacy of Chinese medicine in alleviating symptoms at the end of three full days of treatment, as well as the cure rate of red and sore throat, stuffy and runny nose, and sneezing, QFDY and low-dose QFDY were superior to the placebo, and the differences were statistically significant (P < 0.01). There was no statistical significance in the comparison between the QFDY group and the low-dose QFDY group (P > 0.05).

3. In terms of the headache cure rate after three full days of treatment, QFDY was superior to the placebo, with a statistically significant difference (P < 0.05), and there was no significant efficacy of low-dose QFDY.

4. Safety comparisons showed no serious adverse events and 30 minor adverse events, which were not clinically considered to be related to the drug and were not statistically significant.

Conclusions: In the treatment of patients with influenza and URTIs caused by PHTS, which are mainly characterized by clinical symptoms such as red and sore throat, stuffy and runny nose, and sneezing, when fever is not obvious or low-grade fever is present, the use of low-dose QFDY to simply alleviate the clinical symptoms is recommended and preferred. Moreover, with its good safety profile, QFDY can be used in the treatment of patients with influenza and URTIs caused by PHTS, which can effectively shorten the duration of fever, significantly increase the total efficacy of Chinese medicine in alleviating symptoms after 3 days of treatment, and accelerate the recovery of symptoms such as red and sore throat, stuffy and runny nose, sneezing, and headache, etc.

Clinical Trial Registration: http://www.chictr.org.cn. Trial number: ChiCTR2100043449. Registered on 18 February 2021.

## INTRODUCTION

QFDY, a kind of Chinese herbal medicine preparation, was developed by Professor Guoqiang Mei, under the guidance of the theory of epidemic disease in Chinese medicine. QFDY was created by adding and subtracting the classic prescription Xiao Chaihu Tang and Dayuan-Yin [1], and it was included in the “Chinese Medicine Diagnostic and Treatment Program for New Coronavirus Pneumonia of Hubei Province” by Health Commission of Hubei Province in 2021. It has been recommended by the Hubei Provincial Command of the Prevention and Control of the New Coronavirus Pneumonia Epidemic and approved by the Medical Products Administration of Hubei Province (Z20200003). The quality control standard for the production of QFDY has already been established, and it has been widely used in the clinical treatment of patients with COVID-19 experiencing fever, cough, and fatigue. Recent clinical, randomized, double-blind, placebo-controlled, multicenter trials have demonstrated the therapeutic effect of QFDY on influenza and URTIs caused by PHTS for the first time [2]. At present, QFDY has obtained the approval for registration of preparations for medical institutions (Z20240001) [3, 4].

Traditional Chinese medicine (TCM)-formulated granules are pure Chinese medicine products made from single TCM tablets through extraction, concentration, drying and granulation in accordance with the standard of concoction. TCM tablets and TCM-formulated granules are complementary to each other, with the former being the foundation and the latter being the development. TCM-formulated granules are an important product of the reform of TCM tablets in the 21st century, and they have been gradually applied in the treatment of various diseases. For TCM tablets, it is not easy for people to master its complicated operation method, and it is not possible to guarantee that the composition of the drug is the same every time it is decocted, and many of the substances in it change, making its specific composition more complicated. TCM-formulated granules can effectively avoid the direct effect of incorrect operation of decocting method, so it has a clearer mechanism than that of TCM decoction, at the same time, TCM-formulated granules are available through film coat, or added excipients to improve the stability of drugs, mask uncomfortable odor and slow release and controlled release. TCM-formulated granules not only maintain the advantages of rapid absorption and rapid efficiency of the decoction, but also overcome the need for temporary decoction, long time consumption, easy deterioration, and other shortcomings. The development of TCM-formulated granules maximizes efficacy; besides, advanced scientific extraction methods and equipment make the quality of Chinese medicinal materials relatively guaranteed, the active component is relatively stable, all quality indicators are easy to control and monitor, and production is more scientific, standardized and international [5, 6]. Although there are many comparative studies in terms of ingredient dissolution, pharmacological effects, and clinical efficacy, there is no recognized evaluation method or evaluation standard for the consistent efficacy of formulated granules and traditional decoction. However, in most clinical and experimental studies, the clinical efficacy and active ingredients of TCM-formulated granules have been shown to be equal to or higher than those of TCM tablets, however, the price of TCM-formulated granules is high, and this problem makes TCM-formulated granules inconvenient for patients while increasing their medical burden [7].

In addition, we oppose the reckless use of large doses without regard to a patient’s condition and support the rational use of a large or small dose of medication according to a patient’s condition. Based on this, QFDY granules are a TCM-formulated granule preparation that has been put into production and clinically proven to have precise efficacy. We now put forward the idea that, considering TCM formula granule preparation, a reduced dose of QFDY may also be effective in the treatment of influenza and URTIs caused by PHTS. Therefore, we designed a smaller dosing schedule to further investigate the differences in efficacy of the low-dose QFDY treatment, with half the conventional dose, to ensure the accuracy of clinical dosing and the rational use of TCM-formulated granules with the aim of providing evidence-based medicine with higher clinical value and review of the level of application for the further development and application of QFDY [8].

## MATERIALS AND METHODS

### Research design

**Figure 1:**
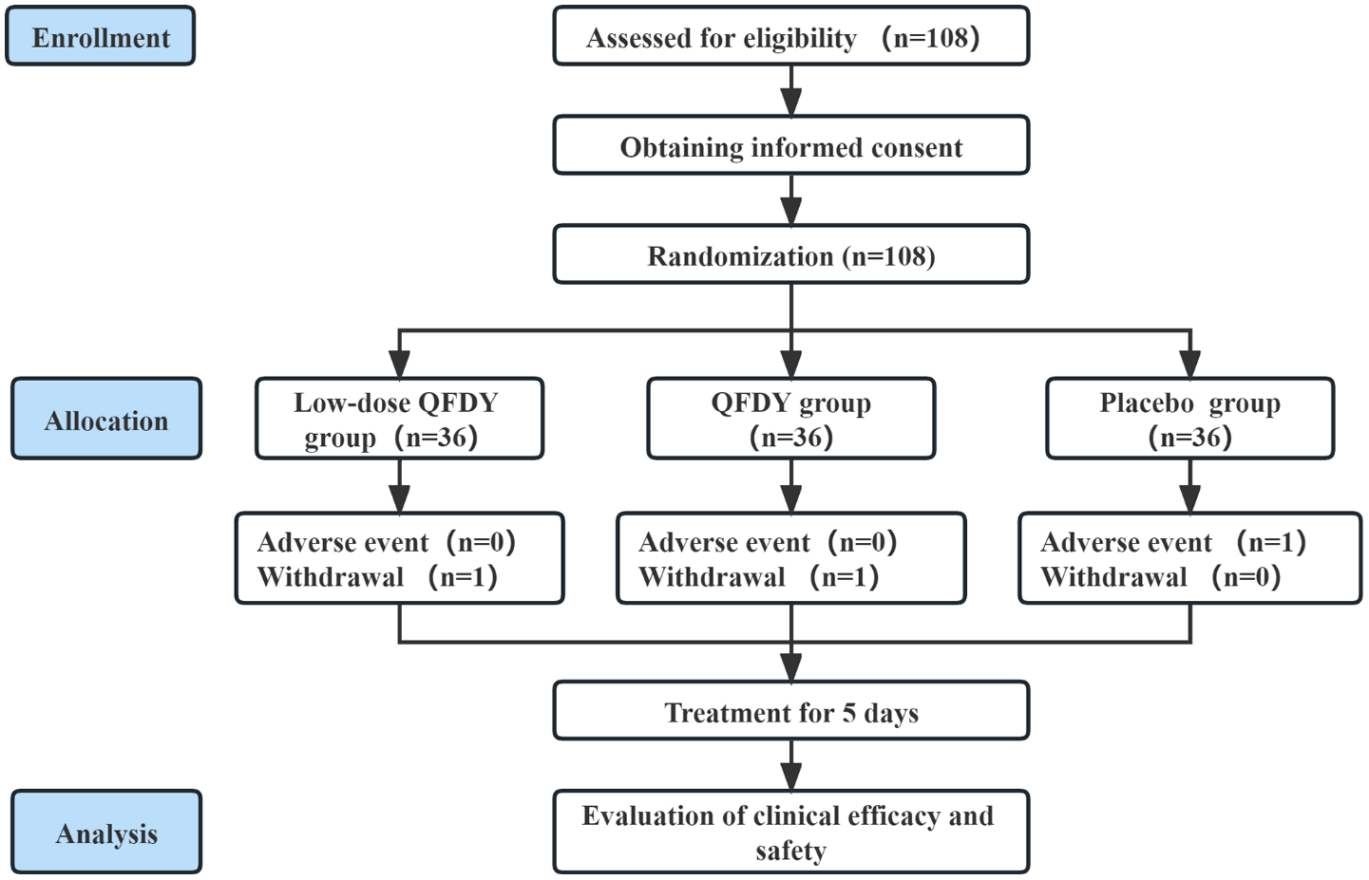
Flow diagram of the study.

Subjects were randomly assigned to three groups of equal size to receive daily oral QFDY (n = 36), low-dose QFDY (n = 36), and placebo (n = 36). All patients received 15 g/dose of each drug or placebo 3 times/day. The duration of treatment was 5 ± 2 days in all groups. The drugs were discontinued early if fever resolved completely and the main symptoms subsided (Figure 1).

### Recruitment of participants

The trial ran from July 8, 2022, to January 23, 2023. This study enrolled 108 patients with influenza and URTIs caused by PHTS. Patients were recruited from 6 tertiary-level A hospitals in 4 major cities in Hubei Province. Eligible patients were consecutively enrolled at a 1:1:1 ratio and randomized to receive QFDY, low-dose QFDY or placebo for 5 days; patients were observed for 7 days. Every effort was made to protect the privacy of patients’ medical information to the extent permitted by law. Patients have the right to full privacy, and written informed consent has been obtained for all disclosures.

### Inclusion and exclusion criteria

The relevant criteria are based on *the Diagnosis and Treatment Protocol of Influenza (2020)* [9] *and the Acute Upper Respiratory Tract Infections (practice version 2018)* [10]. Specific criteria are shown in Table 1 [11].

**Table 1:** Inclusion and exclusion criteria.

**Inclusion criteria**	Meeting all of the following criteria:
	• met the clinical diagnostic criteria of influenza or URTIs;
	• met the differentiation criteria of PHTS;
	• aged 18-70 years old;
	• persistent fever ≤ 48 hours with an axillary temperature ≥ 37.3° C;
	• signed informed consent.
**Exclusion criteria**	Patients meeting any of the following were not eligible for participation in this study:
	• severe or critical cases of influenza or a diagnosis of COVID-19, pharyngo-conjunctival fever, herpangina, and/or suppurative amygdalitis;
	• individuals with influenza comorbidity;
	• taking anti-influenza medication 48 hours before diagnosis;
	• having received systemic therapy with steroids or other immunosuppressants;
	• having a history of seizures or febrile convulsions;
	• with an underlying systemic primary disease;
	• pregnancy or breastfeeding, body sensitivities, allergies to multiple medications or to some of the known ingredients of the medications used in this study;
	• other factors affecting follow-up

### Drugs

The main components of QFDY are shown in Table 2. The drug is manufactured by *Jing Brand Chizhengtang Pharmaceutical Co., Ltd.* (Huangshi City, Hubei Province, China) under strict control in accordance with *Good Manufacturing Practices* (GMP) standards. The active ingredient of low-dose QFDY was half of that of QFDY. Quality control analysis of QFDY has been carried out using ultra performance liquid chromatography (UPLC) technology [12, 13], and the production of its placebo is also handled by the same company, with the production process ensuring that QFDY and its placebo are as similar as possible in terms of color, odor, taste, appearance and packaging. (Supplementary Material 1).

**Table 2:** QFDY drug composition.

**Latin name**	**Genus (taxonomy)**	**Chinese name**	**Part used**	**Herb medicine (g)**	**Entry of Chinese medicine granules into medicine (g)**
*Bupleuri radix*	*Apiaceae*	Chaihu	Dry Root	20	5.81
*Scutellariae Radix*	*Lamiaceae*	Huangqin	Root	10	2.90
*Pinelliae rhizoma praeparatumThunb*.	*Araceae*	Banxia	Tuber	10	2.90
*Codonopsis Radix*	*Campanulaceae*	Dangshen	Dry Root	15	4.35
*Arecae Semen*	*Arecaceae*	Binglang	Seeds	10	2.90
*Tsaoko fructus*	*Zingiberaceae*	Caoguo	Fruits	15	4.35
*Trichosanthis fructus*	*Cucurbitaceae*	Gualou	Fruits	10	2.90
*Magnoliae officinalis cortex*	*Magnoliaceae*	Houpu	Root bark	15	4.35
*Anemarrhenae rhizomaRhizoma*	*Asparagaceae*	Zhimu	Rootstock	10	2.90
*Paeoniae radix rubra*	*Paeoniaceae*	Chishao	Rootstock	10	2.90
*Glycyrrhizae radix et rhizoma*	*Fabaceae*	Gancao	Rootstock	10	2.90
*Citri reticulatae pericarpium*	*Rutaceae*	Chenpi	Exocarp	10	2.90
*Polygoni cuspidati rhizoma et radix*	*Polygonaceae*	Huzhang	Rootstock	10	2.90

### Randomization and blinding

A block group randomization method was used, and the subjects were divided into the QFDY group, low-dose QFDY group and placebo control group at a ratio of 1:1:1. The SAS statistical software Proc Plan process was utilized to generate a randomized arrangement of the treatments received by each of the 108 subjects, given the number of seeds. There was competition among subcenters for enrollment in the groups. The test drug and the simulant of the test drug were produced and packaged by the sponsor, and the test drug and simulant and their packaging were indistinguishable from each other in terms of appearance, thereby meeting the requirements of blinding. The placebo was identical to its simulant except that it did not contain the active ingredient.

### Concomitant medication

During the course of this study, the use of any medication other than the three groups of therapeutic drugs was prohibited. However, patients could be treated with acetaminophen (≤ 500 mg/time; q.i.d.) when they had a prolonged hyperthermia, as well as with antimicrobials when they had a bacterial infection, and all medications used needed to be recorded.

### Outcome measures

Outcome measures are shown in Table 3.

**Table 3:** Outcome measures.

**Primary effectiveness outcome**	The time to complete fever relief (hours).
**Secondary effectiveness outcomes**	• therapeutic effect of TCM on syndromes (effective rate);
	• cure rates for each single symptom, time to cure and remission of symptoms, were recorded on log cards at baseline, at the end of treatment, and in clinical studies, as well as assessments of treatment endpoints;
	• incidence of comorbidities and progression to severe cases as well as the assessments of treatment endpoints;
	• viral antigen-negative conversion rates at baseline and testing at the end of treatment, as well as assessment of treatment endpoints, including influenza virus antigen testing (IV-A + IV-B), antibody testing for coxsackie virus, syncytial virus, ADV, and PIV, and serologic testing for other viruses;
	• laboratory test results, including routine blood tests, c-reactive protein levels, and calcitonin levels, at baseline and after stopping treatment.

### Endpoint definitions

Time to complete antipyretic (hours), axillary temperature was measured every 6 h after the first dose of the drug, and the treatment endpoint was evaluated.

Definition of complete antipyretic: after treatment, axillary temperature < 37.3° C, and maintained for more than 24 h. The treatment endpoint was defined as the time to complete antipyretic.

See Supplementary Materials 2, 3 for specific efficacy evaluation criteria.

### Safety evaluation

The main objective of safety evaluation is to identify significant risk events such as suspected and unanticipated serious adverse reactions in a timely manner through continuous monitoring, real-time analysis and assessment of safety data, and to take appropriate measures to adequately control the risks. It mainly includes: adverse events (AE), serious adverse events (SAE), and clinically significant changes in vital signs or examination data.

### Sample size and statistical analysis

Using the main time to fever relief as the basis for estimation of the sample size, using PASS15.0 software, based on the sample size calculation for the comparison of the sample means of multiple groups, the results of the previous study showed that the mean time to fever relief in each group was 30 h, 36 h, and 40 h, with a standard deviation of 10. The hypothesis test of the type I error α was set to 0.05, the power was set to 0.9, and the sample size of each group was set at a ratio of 1:1:1. To get the minimum sample size for 3 groups for a total of 27 participants in each group, the missing data rate was set to 10%, then the number of people in each group was set to 30, taking into account that the data may have a nonnormal distribution, and then the sample size was increased by 20%, with 36 people in each group, for a total of 108 people.

The study was statistically analyzed using the SPSS 23.0 statistical software package.

Mean ± standard deviation/median (p25, p75) was used to statistically describe the measurement data, and frequencies were used to describe the count and ordinal data.

For measurement data, F-test and Kruskal-Wallis H-test were used for comparison between groups, and chi-square test and Fisher’s exact test were used for comparison of count data between groups.

p-values were adjusted for multiple testing by Bonferroni correction, and the study satisfied the principle of ITT analysis.

Statistical tests were performed using two-sided tests, with the significance level set at 0.05 (P = 0.05).

## RESULTS

In this study, 108 subjects were screened from 6 clinical trial centers from July 2022 to January 2023, of which 36 were included in the QFDY group; 1 patient (2.78%) had shedding, 0 were excluded, and 35 patients completed the trial (97.22%). There was a total of 35 cases in the FAS, 35 cases in the PPS, and 34 cases in the SS in the QFDY group. A total of 36 patients were included in low-dose QFDY group, with 1 patient with shedding (2.78%), 0 patients excluded, and 35 patients completing the trial (97.22%). There was a total of 35 cases in the FAS, 35 cases in the PPS, and 34 cases in the SS in the low-dose QFDY group. A total of 36 patients were included in the placebo control group, with 0 patients with shedding, 0 patients excluded, and 36 patients completing the trial (100%). The difference in the rate of shedding among the three groups was not statistically significant (p > 0.05).

### Baseline characteristics

There was no statistically significant difference in pre-treatment baseline levels among the three groups of patients in the FAS. (*P* > 0.05) (Table 4).

**Table 4:** Descriptive statistical analysis (FAS).

**Variables**	**Full analysis set**				**P-Value**
	**Total (n=106)**	**QFDY group (n=35)**	**Low-dose QFDY group (n=35)**	**Placebo group (n=36)**	
Age (year)^a^	39.1 (18,66)	36.9 (19,60)	40.1 (18,66)	39.8 (21,65)	0.531
Males^b^	49 (46.2)	15 (42.9)	17 (48.6)	17 (47.2)	0.912
Minority^b^	9 (8.5)	1 (2.9)	5 (14.3)	3 (8.3)	0.237
BMI^a^	22.5 (2.745)	22.7 (3.089)	22.4 (2.772)	22.3 (2.404)	0.808
Course of disease (hours)^a^	19.24 (1,48)	21.69 (2,48)	17.17 (1,48)	18.86 (1,40)	0.307
Highest body temperature 48 hours before diagnosis (° C)^a^	38.1 (37.5,40)	38.1 (37.5,39)	38.1 (37.5,40)	38.1 (37.5,39.8)	0.696
Etiological testing of swabs					
Coxsackievirus (positive)^b^	2 (1.9%)	1 (2.9%)	0 (0)	1 (2.8%)	0.544
EB virus (positive)^b^	10 (9.4%)	5 (14.3%)	3 (8.6%)	2 (5.6%)	0.434
Syncytial virus (positive)^b^	0 (0%)	0 (0%)	0 (0%)	0 (0%)	-
ADV (positive)^b^	0 (0%)	0 (0%)	0 (0%)	0 (0%)	-
PIV (positive)^b^	12 (11.3%)	1 (2.9%)	5 (14.3%)	6 (16.7%)	0.309
Total score of TCM symptoms^a^	15.8 (6,31)	15.7 (6,25)	15.3 (8,31)	16.4 (8,27)	0.559

^a^Number, mean, standard deviation.

^b^Number, percentage.

^∆^Fisher’s Exact Test.

### Primary outcome measures

**Figure 2:**
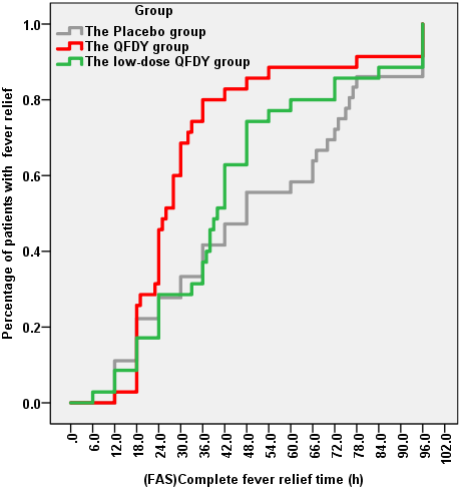
Time to complete fever relief.

**Figure 3:**
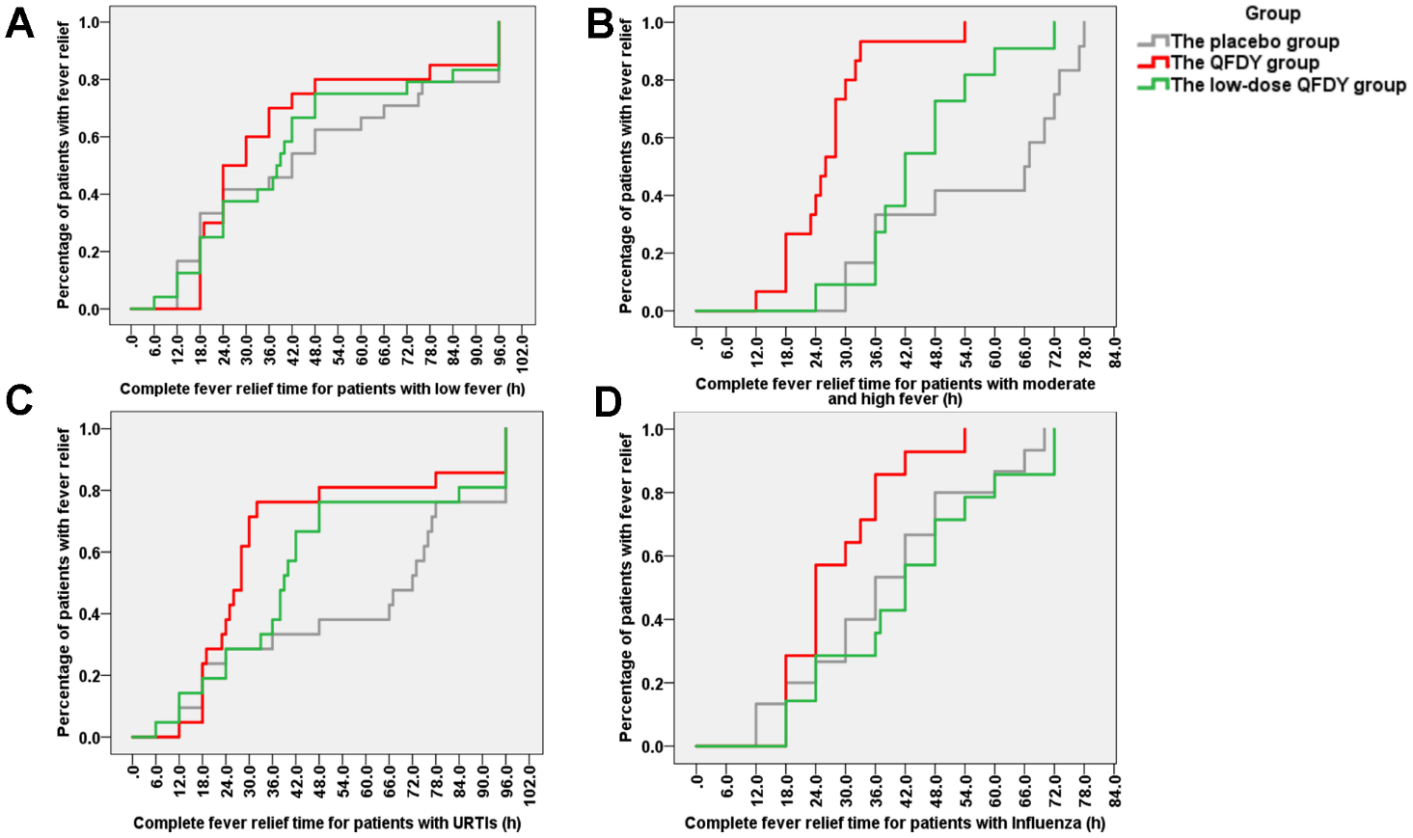
**Stratification comparing the groups according to the time to complete fever relief.** (**A**) Proportion of patients with fever in the three groups after treatment with drugs for low fever (37.3° C -38° C); (**B**) Proportion of patients with fever in the three groups after treatment with drugs for moderate or high fever (38.1° C-41° C); (**C**) Proportion of patients with fever in the three groups after treatment with drugs for URTIs; (**D**) Proportion of patients with fever in the three groups after treatment with drugs for influenza.

The primary outcome was the time to complete fever relief. In the FAS, the QFDY group required 26 hours (12.0, 96.0) for complete fever relief, which was significantly shorter than the time of 40 hours (6.0, 96.0) in the low-dose QFDY group and 48 hours (12.0, 96.0) in the placebo group. According to the Kaplan-Meier curve (Figure 2), both QFDY groups showed better fever relief effects than the placebo group, where the difference was statistically significant in the QFDY group compared to the placebo group (p < 0.05). In response to the different body temperatures of the patients before treatment, 106 patients were classified as having low fever (37.3-38° C), and 69 and 37 patients were classified as having moderate and high fever (38.1-41° C), respectively. According to the Kaplan-Meier curves (Figure 3A), the three groups did not have a significant efficacy in terms of the time to complete fever relief compared with patients with low fever; however, according to the Kaplan-Meier curve (Figure 3B), the two different QFDY dosage groups showed significant superiority in terms of time to complete fever relief for patients with moderate and high fever, in which the QFDY group showed significant superiority compared to the low-dose QFDY group. According to the different diseases of the patients, the statistical results showed that 43 patients were diagnosed with influenza and 63 patients were diagnosed with URTIs by the clinicians, and according to the Kaplan-Meier curve (Figure 3C), the two different QFDY dosage groups showed a better fever relief effect than the placebo group in the treatment of patients with URTIs; moreover, the QFDY group showed a better antipyretic effect than the other two groups according to the Kaplan-Meier curve (Figure 3D). The QFDY group showed a better antipyretic effect than the other two groups in the treatment of patients with influenza; however, the low-dose QFDY group showed no significant efficacy compared with the placebo group.

### Secondary outcome measures

Secondary outcomes were analyzed as follows (Table 5):

**Table 5:** Analysis of secondary outcomes (FAS).

**Variables**	**Full analysis set**		
	**QFDY group (n=35)**	**Low-dose QFDY group (n=35)**	**Placebo group (n=36)**
**Efficacy on TCM symptoms**			
**Three-day treatment**			
Clinical recovery^b^	6 (17.1%)^*^	6 (17.1%)^*^	0 (0%)
Markedly effective^b^	14 (40%)^*^	15 (42.9%)^*^	4 (11.1%)
Effective^b^	14 (40%)^#^	12 (34.3%)^#^	23 (63.9%)
Ineffective^b^	1 (2.9%)^*^	2 (5.7%)^#^	9 (25%)
**Five-day treatment**			
Clinical recovery^b^	26 (74.3%)	22 (62.9%)	25 (68.4%)
Markedly effective^b^	6 (17.1%)	10 (28.6%)	9 (25%)
Effective^b^	3 (8.6%)	3 (8.6%)	2 (5.6%)
Ineffective^b^	0 (0%)	0 (0%)	0 (0%)
**Scores of TCM symptoms**			
Before treatment^a^	15.89 (8,27)	15.33 (6,26)	15.89 (8,31)
Three-day treatment^a^	6.28 (0,24)	6.07 (0,14)	6.28 (2,25)
Five-day treatment^a^	1.25 (0,8)	1.24 (0,7)	1.26 (0,7)
**Cure rate of each single symptom (after a 3-day treatment regimen)**			
Cough^b^	23 (65.7%)	18 (51.4%)	23 (63.9%)
Red and sore throat^b^	8 (22.9%)^*^	14 (40%)^*^	33 (91.7%)
Muscular soreness^b^	5 (14.3%)	5 (14.3%)	7 (19.4%)
Headache^b^	4 (11.4%)^#^	5 (14.3%)	12 (33.3%)
Stuffy and runny nose, sneezing^b^	9 (25.7%)^*^	13 (37.1)^*^	36 (100%)
Expectoration^b^	17 (48.6%)	14 (40%)	15 (41.7%)
Thirst^b^	11 (31.4%)	7 (20%)	9 (25%)
Poor appetite^b^	8 (22.9%)	7 (20%)	7 (19.4%)
Red eyes^b^	2 (5.7%)	1 (2.9%)	0 (0%)
Constipation^b^	3 (8.6%)	2 (5.7)	2 (5.6)
**Incidence of comorbidities^b^**	0 (0%)	0 (0%)	0 (0%)
**Incidence of progression to severe conditions^b^**	0 (0%)	0 (0%)	0 (0%)
**Concomitant Medication**			
Acetaminophen tablets^b^	0 (0%)	2 (5.7%)	3 (8.3%)
Within 24 hours (300-500 mg)^b^	0 (0%)	1 (2.9%)	1 (2.8%)
Within 24-72 hours^b^	0 (0%)	1 (2.9%)	2 (5.6%)
Antibiotics^b^	2 (5.7%)	4 (11.4%)	5 (13.9%)
Within 24 hours^b^	0 (0%)	1 (2.9%)	2 (5.6%)
More than 24 hours^b^	2 (5.7)	3 (8.6%)	3 (8.3%)
Phlegm-resolving medicines^b^	0 (0%)	0 (0%)	0 (0%)

^a^Number, mean, standard deviation.

^b^Number, percentage.

^∆^Fisher’s Exact Test.

Note: Compared with placebo group, * P < 0.01 ^#^ P < 0.05.

**Figure 4:**
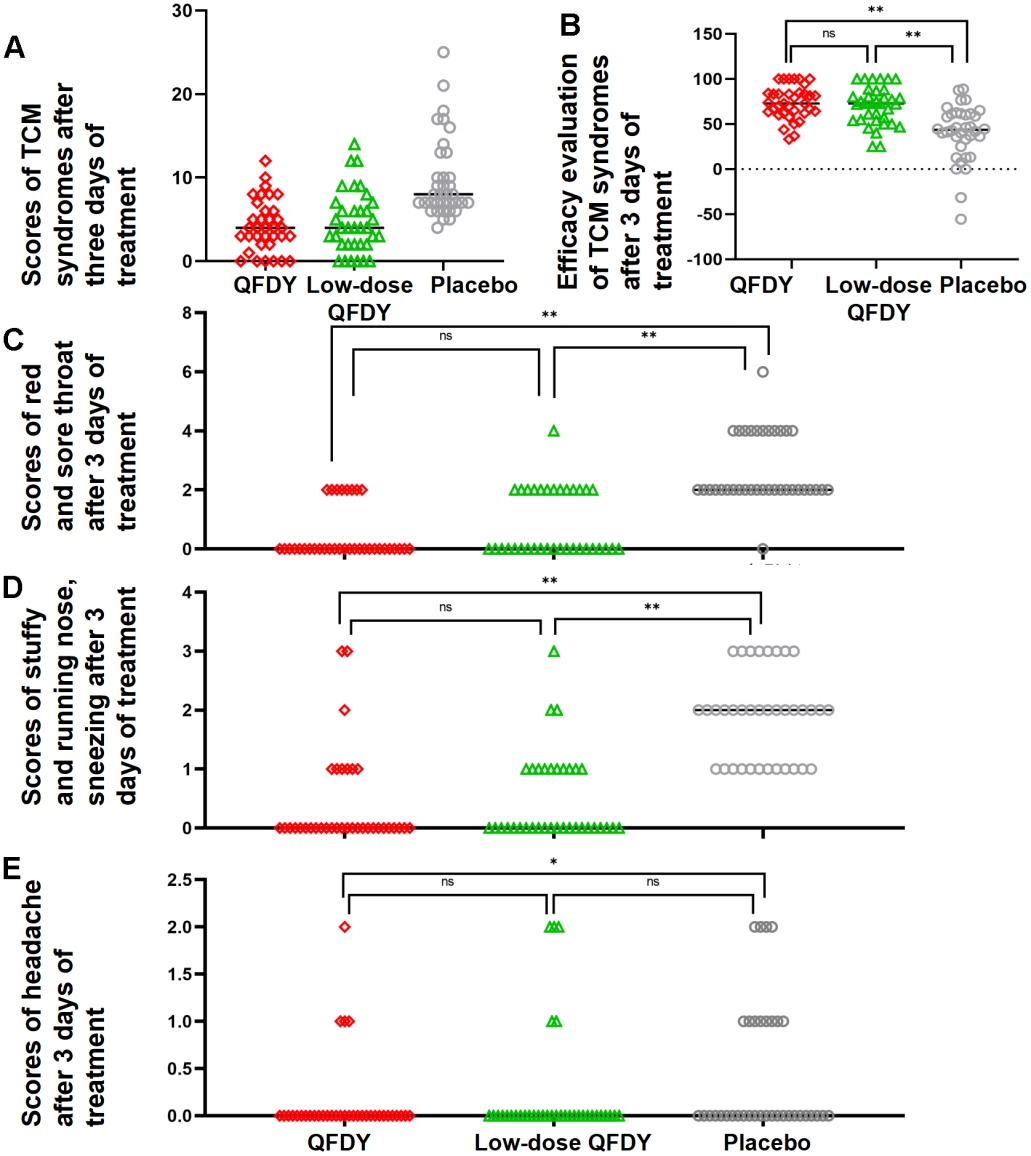
**Scatterplot of results for secondary efficacy indicators.** (**A**) Scatterplot of scores of TCM symptoms after 3 days of treatment; (**B**) Scatterplot of efficacy evaluation of TCM symptoms after 3 days of treatment; (**C**) Scatterplot of scores of red and sore throat after 3 days of treatment; (**D**) Scatterplot of scores of stuffy and runny nose and sneezing after 3 days of treatment; (**E**) Scatterplot of scores of headache after 3 days of treatment.

1. Efficacy evaluation of TCM symptoms: After 3 days of treatment, compared with the placebo group, the QFDY and low-dose QFDY groups showed statistically significant differences (P < 0.05) in clinical recovery, marked effectiveness, effectiveness and ineffectiveness in the FAS, but there was no significant difference in the clinical recovery, marked effectiveness, effectiveness and ineffectiveness of the three groups after 5 days of treatment (P > 0.05). In summary, the results showed that both QFDY doses were significantly better than the placebo in terms of the efficacy evaluation of TCM symptoms after 3 days of treatment (Figure 4B).
2. Symptom disappearance rate: After 3 days of treatment, the efficacy of the QFDY group and the low-dose QFDY group was comparable in terms of the disappearance rate of red and sore throat, stuffy and runny nose, sneezing, and the difference was not statistically significant (P > 0.05) (Figure 4C, 4D). The disappearance rate of headache after 3 days of treatment was better in the QFDY group than in the placebo group, and the difference was statistically significant (P < 0.05) (Figure 4E).
3. Scores of TCM symptoms: In the FAS, after the full 3 days of treatment, the efficacy of the low-dose QFDY group and the QFDY group was comparable relative to the placebo group (p < 0.05) (Figure 4A).
4. Complications and the development of serious conditions were not observed in any of the three groups.
5. Concomitant medication: There was no statistically significant difference between the three groups in terms of concomitant medications (P > 0.05).
6. Laboratory tests: There were no significant differences among the three groups (P > 0.05).

### Safety analysis

Analysis of SS revealed that no serious adverse events occurred in this study. Among them, mild adverse events occurred in 30 patients, as follows:

1. QFDY group: 9 adverse events occurred in 8 patients, including 2 cases of elevated serum aminotransferase level, 4 cases of abnormal urine test, and 3 cases of positive fecal occult blood (FOB).
2. Low-dose QFDY group: 14 adverse events occurred in 13 patients, including 5 cases of elevated serum aminotransferase, 8 cases of abnormal positive urine test, and 1 case of positive FOB.
3. The placebo group, 10 adverse events occurred in 9 patients, including 3 cases of elevated serum aminotransferase levels, 6 cases of abnormal urine tests or mild serum creatinine abnormalities (Scr), and 1 case of positive FOB.

## DISCUSSION

We advocate that large doses of QFDY can be used for specific conditions—acute, critical, and severe—and specific stages—acute attacks—to contain diseases and control conditions, and QFDY has a rapid onset of action, in which the dose is reduced, e.g., by 1/2 or ¼, for patients in the middle of the disease. The key to the so-called rational use of medication lies in the condition, emphasizing that the use of a large dose should not ignore the corrective effect of a small dose based on low evidence and should oppose a general increase in the dose without regard to the patient condition. In view of this, this study, using QFDY as the main treatment, used statistical methods and intergroup comparisons to explore the efficacy of different dosages of QFDY treatment for influenza and URTI patients to guide the rational clinical dosage and to ensure the rational use of traditional Chinese medicine formulated particles to improve clinical efficacy.

QFDY is a formula that originated from Prof. Mei Guoqiang, a typhoid scientist, and is tested formula for treating PHTS during COVID-19. It is effective in reconciling Shaoyang, resolving dampness and performing detoxification and is mainly used to treat fever, headache, stuffy and runny nose, sneezing, sore throat, general fatigue, muscular soreness, cough and sputum caused by PHTS. The 13 medicines in QFDY did not show any significant toxic effects in pre-acute toxicology and long-term toxicity tests, indicating that the formulation has a high safety profile. The effectiveness of QFDY has also been preliminarily confirmed in previous clinical trials. The present study aimed to conduct a phase IIb clinical trial on the basis of the previous phase IIa clinical trial to do further comparison of the differences in efficacy between the three groups of drugs.

The results of the study showed that QFDY could dose-dependently shorten the duration of fever in patients with influenza and URTIs in terms of the time to complete fever relief, and the Kaplan-Meier estimation curves for the duration of antipyretic use at each time point in the three groups also visually demonstrated that QFDY significantly shortened the time to complete fever relief, and low-dose QFDY also shortened the duration of fever relative to the placebo, although the difference between the groups was not statistically significant. Regarding the cure rate of each single symptom, both QFDY doses could effectively shorten the disappearance time of all symptoms, especially the disappearance time of red and sore throat, stuffy and runny nose, and sneezing. In terms of clinical efficacy, both QFDY dosages were able to significantly increase the efficacy of TCM symptom reduction for patients with influenza and URTIs caused by PHTS compared with the placebo (P < 0.05), and both QFDY dosages were able to improve the scores of TCM symptoms (P < 0.05), which were lower than those of the placebo at the end of the third day of treatment. Symptoms were relieved after 5 full days of treatment, and the difference between the 3 groups was not statistically significant. Therefore, early use of QFDY within 3 days of diagnosis may help to achieve more pronounced efficacy. This study stratified patients by disease and temperature categories and found that in the treatment of patients with moderate to high fever (38.1-41° C), QFDY had the advantage of significantly shortening the duration of fever, with patients’ fevers starting to lower after 12 h of drug administration, compared to after 24 h of low-dose QFDY administration. In addition, in the treatment of patients with influenza, there was no therapeutic advantage of low-dose QFDY compared to the placebo. In addition, in the treatment of influenza patients, low-dose QFDY had no therapeutic advantage over the placebo, whereas QFDY significantly shortened the duration of fever.

In summary, two points of reflection from this pilot study are provided as follows.

### “Dosage-efficacy” relationship

In this study, only QFDY was found to be significantly effective in relieving headache symptoms, which may be because the QFDY dosage needs to reach a certain level to exert its therapeutic effect in treating headache. Further studies are needed to reveal the specific mechanism of its efficacy and to confirm its clinical efficacy. In addition, in the treatment of patients with influenza, low-dose QFDY did not have any therapeutic advantage over the placebo, while QFDY showed significant efficacy in shortening the duration of fever, which may be because the low dose of QFDY did not achieve therapeutic efficacy in treating influenza, and further well-designed experiments are needed to explore the potential therapeutic benefit of the dose-effect relationship for Chinese herbal medicines. Changes in the dosage of TCM prescriptions are an important tool for guiding clinical practice, and dosage strategy is an important part of the dosage theory of TCM prescriptions. Therefore, we should actively explore new ideas and methods for researching the dose-efficacy relationship for TCM, and on the basis of strict adherence to the law of prescription and dosage, we should carefully follow the pathogenesis of the disease and master the characteristics of the dose-efficacy relationship for TCM so that we can give full play to its maximal clinical value and provide more favorable references for the treatment of a variety of diseases. Further study is needed to maximize the clinical value and provide more favorable references for the treatment of various diseases.

### “Cost-utility” relationship

Our results showed that the therapeutic advantages of QFDY were more obvious for patients with moderate or high fever, whereas low-dose QFDY was effective at low doses for patients with no fever and low fever, or purely for the relief of clinical symptoms. From the perspective of Chinese medicine granules, with the strict requirements of the preparation process and the strict control of the production process, this study showed that lowering the dose of QFDY can still effectively exert a therapeutic effect, which is conducive to greatly reducing the medical burden of patients, and it provides a clinical basis for further exploring the cost-utility comparison of Chinese medicine granules. Currently, Chinese medicine granules are not only widely used in domestic hospital clinics but have also entered the international market, which has enabled the promotion of Chinese medicine culture and the advancement of traditional Chinese medicine [14]. In the future, along with the continuous optimization of extraction, purification and separation and the drying technology for Chinese medicine granules, the advantages of Chinese medicine granules will be further reflected.

### Limitations

Although this study was completed valuably, limitations were still considered. Since statistical tests usually require a larger sample size to ensure the representativeness of the overall distribution, and this study enrolled a total of 108 cases, which is a small sample size, the sample size could be enlarged by recruiting more patients with influenza and URTIs caused by PHTS, which would facilitate the finding of significant relationships from the data of a larger sample size.

## Supplementary Material

Supplementary Material 1

Supplementary Materials 2 and 3
