# Supplementary material for: Exploration of the utility of different doses of Qingfei Dayuan granules: a multicenter, randomized, double-blind, placebo-controlled trial
Source: Aging (Albany NY). 2024 Feb 26;16(5):4503–17. doi: 10.18632/aging.205601 (PMC10968682; doi:10.18632/aging.205601)
Supplement: Supplementary Material 1 [file aging-16-205601-s001.docx]

**Supplementary Material 1:**

**Preliminary study on Qingfei Dayuan granules**

The following studies had been completed. First, the prescription quantity and preparation method of Qingfei Dayuan granules (QFDY) were determined, with a drug-extract ratio of 20.46%. Second, combined with Ultra Performance Liquid Chromatography (UPLC), the main ingredients and contents of each Chinese herbal medicine were identified to follow the requirements of the Pharmacopoeia of the People’s Republic of China. Third, by means of UPLC, 12 compounds with high content of QFDY were identified as the main chemical constituents by comparison with the standard reference, including: arecoline hydrobromide, mangiferin, paeoniflorin, liquiritin, hesperidin, lobetyolin, resveratrol; baicalin, ammonium glycyrrhetate, emodin, honokiol, and magnolol. Then, the quality standard of QFDY was established by fingerprint analysis of the multiple batches of extracts. Finally, the toxicity tests of single administration and repeated administrations were conducted respectively, which discovere no abnormal changes in the various physiological indexes of animals, indicating high safety of the preparation.

**1 The prescription quantity and preparation method**

In proportion to the daily dose, the medicinal materials used in the production of QFDY preparations include: *Bupleurum chinense* DC. [Apiaceae; Bupleuri radix] 444g, *Scutellaria baicalensis*Georgi [Lamiaceae; Scutellariae radix] 222g, *Pinellia ternata*(Thunb.) Breit. [Araceae; Pinelliae rhizoma praeparatum] 222g, *Codonopsis pilosula* (Franch.) Nannf. [Campanulaceae; Codonopsis radix] 333g, *Trichosanthes kirilowii* Maxim. [Cucurbitaceae; Trichosanthis fructus] 222g, *Areca catechu* L. [Arecaceae; Arecae semen] 222g, *Lanxangia tsao-ko* (Crevost & Lemarié) M.F.Newman & Škorničk. [Zingiberaceae; Tsaoko fructus] 333g, *Magnolia officinalis* Rehder & E.H.Wilson [Magnoliaceae; Magnoliae officinalis cortex] 333g, *Anemarrhena asphodeloides* Bunge [Asparagaceae; Anemarrhenae rhizoma] 222g, *Paeonia lactiflora* Pall. [Paeoniaceae; Paeoniae radix rubra] 222g, *Glycyrrhiza uralensis* Fisch. ex DC. [Fabaceae; Glycyrrhizae radix et rhizoma] 222g, Citrus × aurantium f. deliciosa (Ten.) M.Hiroe [Rutaceae; Citri reticulatae pericarpium] 222g, and *Polygonum cuspidatum* Siebold & Zucc. [Polygonaceae; Polygoni cuspidati rhizoma et radix] 222g. The above 13 botanical drugs were decocted in water twice. Decoct them 60 minutes in water (volume ratio: 1:8) and 45 minutes (volume ratio: 1:6) respectively. Then vacuum concentration is performed until the concentrated extract has a density of 1.07～1.12 (65℃~75℃). After the dry paste powder was prepared by spray drying, silicon dioxide (1% by weight) and maltodextrin (an appropriate amount) were added successively to until reaching 1000g. The mixture then turns into a granule product through granulation. The drug-extract ratio is 20.46%.

**2 Thin layer chromatography (TLC)**

Uder the quality control, the main indicative ingredients of medicinal materials and their preparations were analyzed by TLC. (Figure 1)

**FIGURE 1** Thin layer chromatography (TLC)


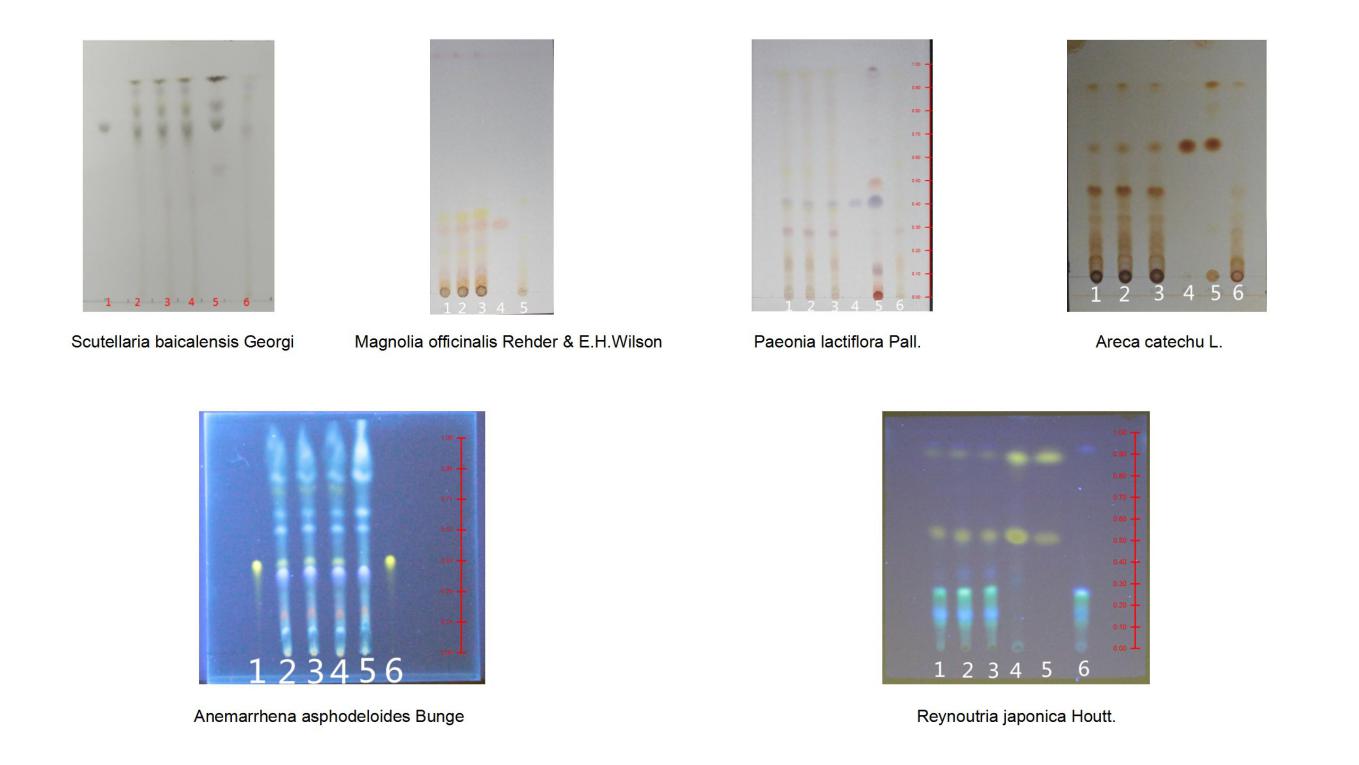


## 3 Content determination

## 3.1 Indicative ingredients

According to the product features, a trial was conducted on 14 indicative ingredients, including Saikosaponin A, Saikosaponin D, baicalin, lobetyolin, Arecoline hydrobromide, magnolol, honokiol, mangiferin, paeoniflorin, liquiritin, ammonium glycyrrhetate, hesperidin, emodin and resveratrol. The reference mixed solution and QFDY solution were injected into the liquid chromatograph respectively in accordance with the standardized methods. The spectrogram showed the higher peak, larger peak area and higher degree of separation of baicalin, hesperidin and paeoniflorin under the selected chromatographic conditions, with desirable effect of separation. By contrast, the separation effect of other ingredients was poor, and the peak and peak area were relatively small. When the retention time of Saikosaponin A is 22.36 minutes and Saikosaponin D is 23.94 minutes, there were few peaks, indicating low content of the two. Therefore, baicalin, hesperidin and paeoniflorin were chosen as the indicative ingredients and their content was determined. The chromatograms of mixed control solution and QFDY solution are as follows. (Figure 2,3)

**FIGURE 2** Chromatogram of mixed control solution

**1**

**2**

**3**

**4**

**5**

**6**

**7**

**8**

**9**

**10**

**11**

**12**

**FIGURE 3** Chromatogram of QFDY Solution

**1**

**2**

**3**

**4**

**5**

**6**

**7**

**8**

**9**

**10**

**11**

**12**

Note: Peak 1-Arecoline hydrobromide; Peak 2-mangiferin; Peak 3-paeoniflorin; Peak 4-liquiritin; Peak 5-hesperidin; Peak 6-lobetyolin; Peak 7-resveratrol; Peak 8-baicalin; Peak 9-ammonium glycyrrhetate; Peak 10-emodin; Peak 11-honokiol; Peak 12- magnolol.

**3.2 Chromatographic conditions and system suitability**

**3.2.1 Selection of detection wavelength (Figure 4-8)**

**FIGURE 4** Full wavelength scanning 3D image of paeoniflorin, hesperidin and baicalin

**FIGURE 5** UPLC of QFDY mixed control solution (210nm)

**FIGURE 6** UPLC of QFDY mixed control solution (230nm)

**FIGURE 7** UPLC of QFDY mixed control solution (250nm)

**FIGURE 8** UPLC of QFDY mixed control solution (300nm)

**3.2.2 Investigation of chromatographic columns (Figure 9-11)**

**FIGURE 9** Chromatographic columns of ACQUITY UPLC® HSS T3 1.8µm

**FIGURE 10** Chromatographic columns of ACQUITY UPLC® BEH C18 1.7µm

**FIGURE 11** Chromatographic columns of ACQUITY UPLC® BEH Shield RP18 1.7µm

**3.3 Durability test**

**3.3.1 Selection of mobile phase proportions**

Gradient elution was performed with acetonitrile and a certain proportion of acid solution. (Figure 12-14)

**FIGURE 12** Chromatogram A of elution analysis

**FIGURE 13** Chromatogram B of elution analysis

**FIGURE 14** Chromatogram C of elution analysis

**3.3.2 Investigation of column temperatures (Figure 15-17)**

**FIGURE 15** Chromatogram of column temperature at 20 ° C

**FIGURE 16** Chromatogram of column temperature at 30 ° C

**FIGURE 17** Chromatogram of column temperature at 40 ° C

**3.3.3 Investigation of flow rates (Figure 18-20)**

**FIGURE 18** Chromatogram of flow rate of 0.2mL/min

**FIGURE 19** Chromatogram of flow rate of 0.3mL/min

**FIGURE 20** Chromatogram of flow rate of 0.4mL/min

**3.4 Investigation of sample extraction methods (Table 1)**

**TABLE 1** Content determination of indicative ingredients with different extractions

| **Extractions** | **Paeoniflorin**  **（mg/g）** | **Hesperidin**  **（mg/g）** | **Baicalin**  **（mg/g）** |
| --- | --- | --- | --- |
| Ultrasonic with methanol, 20min | 2.5828 | 3.2035 | 12.6399 |
| Ultrasonic with ethanol, 20min | 1.7738 | 2.1671 | 5.3883 |
| Ultrasonic with dilute ethanol, 20min | 2.5091 | 3.1329 | 14.1543 |
| Reflux with dilute ethanol, 20min | 2.5126 | 3.1496 | 14.2111 |
| Ultrasonic with dilute ethanol, 10min | 2.5173 | 3.1346 | 14.1885 |
| Ultrasonic with dilute ethanol, 30min | 2.5007 | 3.1207 | 14.1363 |

**3.5 Flow of determination**

**3.5.1 Chromatographic conditions and system suitability test**

Filler: C_18_ (length of column: 100 mm, inner diameter: 2.1 mm, grain size: 1.8 μm); mobile phase A: acetonitrile, mobile phase B: 0.1% phosphoric acid, gradient elution in accordance with the regulations (Table 2); detection wavelength: 230 nm; column temperature: 30℃; flow rate: 0.4 ml/min. Theoretically, the minimum number of plates derived from the peaks of baicalin is 2,800.

**TABLE 2** Regulations of gradient elution

| **Time（minute）** | **Mobile Phase A (%)** | **Mobile Phase B (%)** |
| --- | --- | --- |
| 0～4 | 10→12 | 90→88 |
| 4～11 | 12→20 | 88→80 |
| 11～21 | 20→40 | 80→60 |
| 21～25 | 40→80 | 60→20 |
| 25～29 | 80 | 20 |
| 29～29.01 | 80→10 | 20→90 |
| 29.01～35 | 10 | 90 |

**3.5.2 Preparation of control solution**

Take exact amount of baicalin, paeoniflorin and hesperidin first, then prepare the control solution (155 μg baicalin/ml; 25 μg paeoniflorin/ml; 30 μg hesperidin/ml) with added dilute ethanol.

**3.5.3 Preparation of QFDY solution**

Take 0.1 gram of the powder, put it into a conical flask with stopper, add 10 ml of dilute ethanol, and cover the flask firmly with the stopper. After weighing, ultrasonic processing is performed (power: 250 W, frequency: 40 kHz) for 20 minutes. The powder should be reweighed when becoming cold. Next, add dilute ethanol to compensate for the lost weight, shake up the solution, and obtain the subsequent filtrate after filtration.

**3.5.4 Determination**

Take 1 μl of the control solution and QFDY solution injected into the liquid chromatograph for determination, and record the spectrogram to acquire data.

The content determination of QFDY is specific, stable and repeatable. Its precision and accuracy tests both meet requirements, and the durability of chromatographic conditions is good. Therefore, the HPLC conditions can be used for determination of paeoniflorin, hesperidin and baicalin in QFDY.

**4 Fingerprint chromatography**

A total of 15 batches of QFDY fingerprint were systematically conducted to establish fingerprint chromatography. (Figure 21,22)

**FIGURE 21** Fingerprint overlay of 15 batches of QFDY


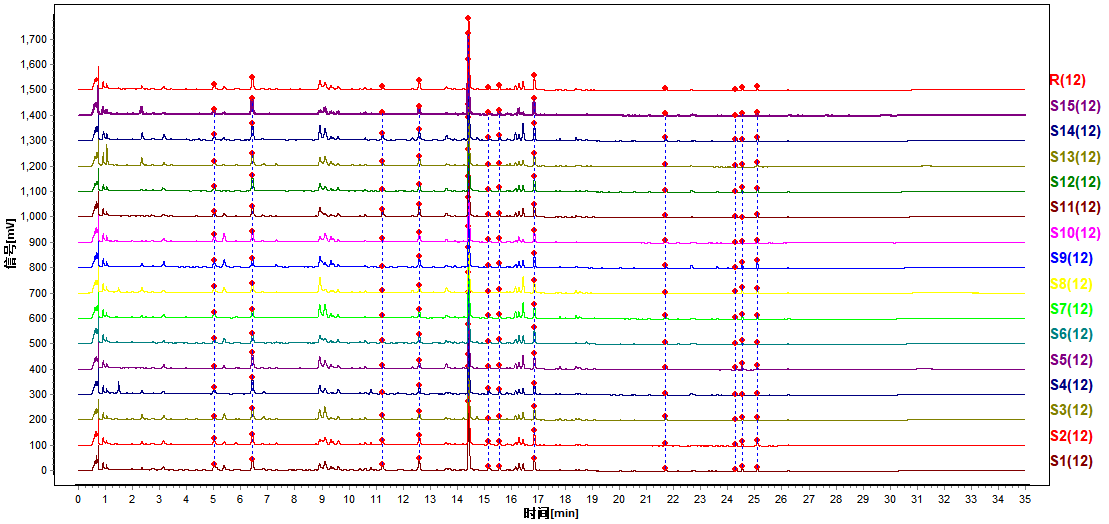


**FIGURE 22** Control fingerprint of 15 batches of sampled QFDY


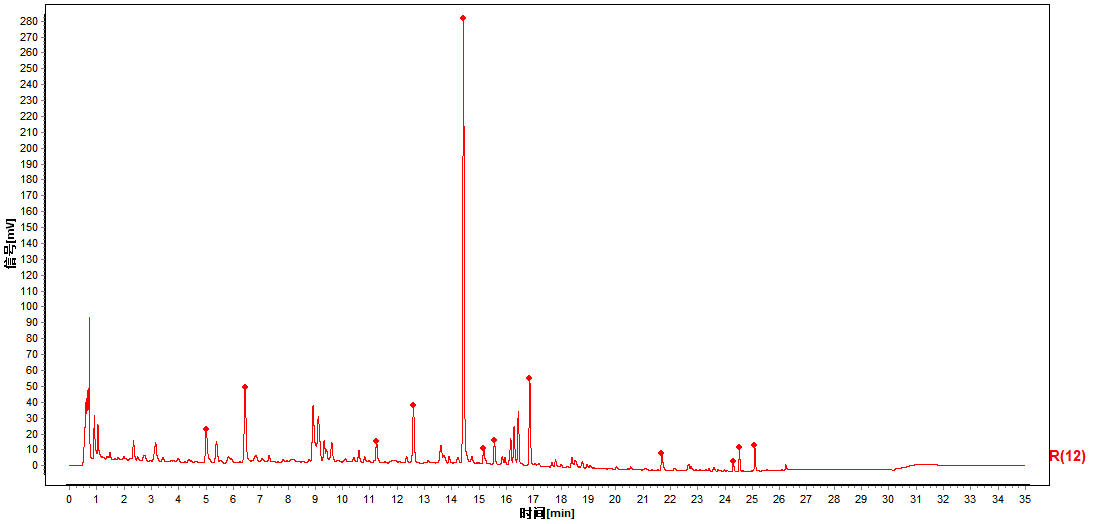


12

11

10

9

8

7

6

5

4

3

2

1

Note: Peak 1-mangiferin; Peak 2-paeoniflorin; Peak 4(s)-hesperidin; Peak 5-baicalin; Peak 9-ammonium glycyrrhetate; Peak 10-emodin; Peak 11-honokiol; Peak 12- magnolol.

**5 Toxicological study**

The toxicity tests of single and repeated administration were performed in laboratories with GLP qualification requirements. The results showed that the maximum tolerable dose (MTD) of rats was greater than 24.0 g/kg/ day (equaling 124.0 g/kg of crude drug) when dry paste powder suspension of QFDY was gavaged twice within 24 hours. The no-observed-adverse-effect level (NOAEL) was 16.0 g/kg/ day (equaling 82.7 g/kg of crude drug) after rats were orally administered with dry paste powder of QFDY for 1 month.
