# Supplementary material for: Exploration of the utility of different doses of Qingfei Dayuan granules: a multicenter, randomized, double-blind, placebo-controlled trial
Source: Aging (Albany NY). 2024 Feb 26;16(5):4503–17. doi: 10.18632/aging.205601 (PMC10968682; doi:10.18632/aging.205601)
Supplement: Supplementary Materials 2 and 3 [file aging-16-205601-s002.pdf]

Supplementary Material 2. Efficacy evaluation criteria.

**Complete fever relief**	An axillary temperature kept below 37.3° C after treatment that lasts for more than 24 hours.
**Overall therapeutic effect on TCM symptoms**	The evaluation criteria for cold in the Criteria of Diagnosis and Therapeutic Effect of Disease and Syndromes in Traditional Chinese Medicine (an industrial standard of TCM of the People's Republic of China) (ZY/T001.1-94) were adopted by referencing the Guidelines for Clinical Research of New Drugs of Traditional Chinese Medicine (trial version) and the Technical Guidelines for Clinical Research of New Drugs of Traditional Chinese Medicine Treating Influenza. The formula for the overall therapeutic effect was as follows: (score before treatment - score after treatment)/score before treatment × 100%; overall effective rate = clinical recovery rate + markedly effective rate + effective rate.<br>• Clinical recovery: TCM symptoms and signs have totally or basically subsided, with the quantitative scores of symptoms showing a decrease of at least 95%.<br>• Marked effectiveness: Clinically, TCM symptoms and signs have been relieved significantly, with the quantitative scores of symptoms showing a decrease of 70% to 95%.<br>• Effectiveness: Clinically, TCM symptoms and signs have been relieved, with the quantitative scores of symptoms showing a decrease of 30% to 70%.<br>• Ineffectiveness: Clinically, no significant improvement of TCM symptoms or signs has been found, with worsening or deteriorating conditions, and the quantitative scores of symptoms showing a decrease of less than 30%.
**Definition of the quantitative scores of TCM symptoms**	The quantitative scores of TCM symptoms were the sum of all symptom scores. The research team created the quantitative rating table for PHTS treated with TCM (Supplementary Material 3).

Supplementary Material 3. Quantitative rating table for PHTS in TCM.

Grading	-	+	++	+++
**Primary symptoms**	**Zero points**	**Two points**	**Four points**	**Six points**
Fever	Highest axillary temperature < 37.3° C 24 hours before diagnosis	Highest axillary temperature at 37.3-37.9° C 24 hours before diagnosis	Highest axillary temperature at 38-39° C 24 hours before diagnosis	Highest axillary temperature > 39° C 24 hours before diagnosis
Cough	No	Occasional cough	Discontinuous cough with no effects on rest and sleep	Frequent cough with effects on rest and sleep
Red and sore throat	No	A little red and slightly uncomfortable throat	Red throat, painful when swallowing	Obvious hyperemia of throat, difficult to swallow
**Secondary symptoms**	**Zero points**	**One points**	**Two points**	**Three points**
Muscular soreness	No	Slight muscular soreness	Muscular soreness	Muscular soreness, inhibited bending and stretching
Headache	No	Slight headache waxing and waning	Severe persistent headache	Obvious headache affecting work
Stuffy and running nose, sneezing	No	Occasionally	Sometimes	Frequently
Expectoration	No	Occasionally	Sometimes	Frequently
Thirst	No	Occasionally	Sometimes	Frequently
Poor appetite	No	Poor appetite with normal food intake	Poor appetite with food intake cut by 1/3	Poor appetite with food intake cut by 1/2 or more
Red eyes	No	Occasionally	Sometimes	Continuously
Constipation	Once a day	Difficult defecation or once every two days	Once every three days	Once every four or more days
**Other symptoms**				
**Tongue**	Red tongue, yellow or greasy coating		Others:	
**Pulse**	Slippery and rapid pulse		Others:	
